# Ultraviolet (UV) and Hydrogen Peroxide Activate Ceramide-ER Stress-AMPK Signaling Axis to Promote Retinal Pigment Epithelium (RPE) Cell Apoptosis

**DOI:** 10.3390/ijms140510355

**Published:** 2013-05-17

**Authors:** Jin Yao, Hui-E Bi, Yi Sheng, Li-Bo Cheng, Ri-Le Wendu, Cheng-Hu Wang, Guo-Fan Cao, Qin Jiang

**Affiliations:** 1 The Affiliated Eye Hospital of Nanjing Medical University, Nanjing 210029, China; E-Mails: bihuie@yahoo.cn (H.-E.B.); shenyi1983@sina.com (Y.S.); caocong1111@163.com (L.-B.C.); wendurile187@yahoo.com.cn (R.-L.W.); tengyu33@yahoo.com (C.-H.W.); caoguofan587@163.com (G.-F.C.); 2 Eye Department, Li-Yang City Hospital of Traditional Chinese Medicine, Li-Yang City 213300, China

**Keywords:** age-related macular degeneration (AMD), UV, ROS, RPE cell apoptosis, ceramide, ER stress and AMPK

## Abstract

Ultraviolet (UV) radiation and reactive oxygen species (ROS) impair the physiological functions of retinal pigment epithelium (RPE) cells by inducing cell apoptosis, which is the main cause of age-related macular degeneration (AMD). The mechanism by which UV/ROS induces RPE cell death is not fully addressed. Here, we observed the activation of a ceramide-endoplasmic reticulum (ER) stress-AMP activated protein kinase (AMPK) signaling axis in UV and hydrogen peroxide (H_2_O_2_)-treated RPE cells. UV and H_2_O_2_ induced an early ceramide production, profound ER stress and AMPK activation. Pharmacological inhibitors against ER stress (salubrinal), ceramide production (fumonisin B1) and AMPK activation (compound C) suppressed UV- and H_2_O_2_-induced RPE cell apoptosis. Conversely, cell permeable short-chain C6 ceramide and AMPK activator AICAR (5-amino-1-β-D-ribofuranosyl-imidazole-4-carboxamide) mimicked UV and H_2_O_2_’s effects and promoted RPE cell apoptosis. Together, these results suggest that UV/H_2_O_2_ activates the ceramide-ER stress-AMPK signaling axis to promote RPE cell apoptosis.

## 1. Introduction

Age-related macular degeneration (AMD) is a progressive degenerative retinal disease and is the leading cause of blindness among elderly people [1]. The precise etiology of AMD is still not fully addressed, although sunlight ultraviolet (UV) exposure and oxidative stress have been proposed [2–4]. Sunlight UV induces reactive oxygen species (ROS) generation to cause oxidative stress, which is now proposed as the major pathological cause of AMD [5].

ROS impairs the physiological functions of retinal pigment epithelium (RPE) cells by causing RPE cell death. Free radicals, such as superoxide, hydroxyl radical and singlet oxygen, as well as non-radical species, such as hydrogen peroxide (H_2_O_2_), are among ROS that cause cell damage under oxidative stress [6]. Oxygen free radicals are highly reactive and have the capacity to damage cellular components, such as proteins, lipids and nucleic acids [7]. The association between oxidative stress and AMD was further supported by clinical trial studies showing a dramatic reduction AMD progression rate in subjects taking antioxidants and zinc-containing supplements [8,9]. Our previous study demonstrated that nerve growth factor (NGF) rescues oxidative stressed RPE cells by restoring mTOR (mammalian target of rapamycin) activation [10]. The mechanism by which ROS induces RPE cell death is not fully addressed.

Ceramide is a well-known cellular mediator of apoptosis [11]. Agents that enhance intracellular ceramide accumulation would provide pro-apoptotic outcomes. Ceramide promotes cell apoptosis through regulating its downstream targets [11]. For example, ceramide activates JNK-dependent cell apoptosis [12]. Further, ceramide is also known to inhibit Akt activation [11]. Interestingly, recent studies have suggested that ceramide activates AMP activated protein kinase (AMPK)-dependent cell apoptosis pathway [13,14]. Studies have shown that UV and H_2_O_2_-induced cell apoptosis involves ceramide production [15] and AMPK activation [16]. However, whether ceramide and AMPK activation are also important for UV and H_2_O_2_-induced RPE cell apoptosis requires further investigation. In the current study, we studied the apoptosis signaling pathway by UV/ROS in cultured RPE cells. We found that UV and H_2_O_2_ activate the ceramide-ER stress-AMPK signaling axis to promote RPE cell apoptosis.

## 2. Results

### 2.1. H_2_O_2_ Activates ER Stress, AMPK and MAPK Signal Pathways in Cultured RPE Cells

**Figure 1:**
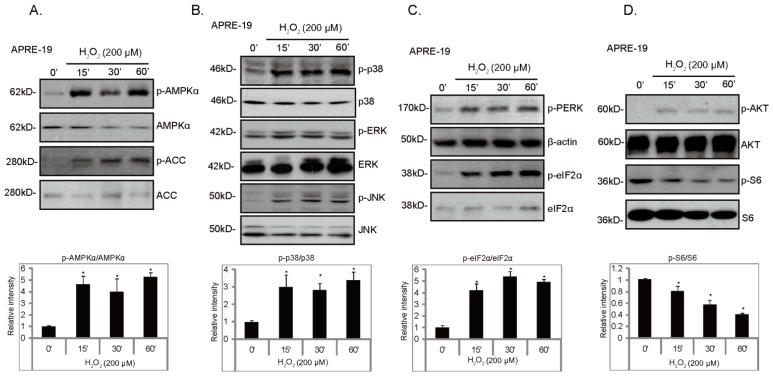
H_2_O_2_ activates endoplasmic reticulum (ER) stress, AMP activated protein kinase (AMPK) and mitogen-activated protein kinase (MAPK) signal pathways in cultured retinal pigment epithelium (RPE) cells. APRE-19 cells were either left untreated or treated with H_2_O_2_ (200 μM) for indicated time points; p-AMPKα (Thr 172) (**A**), p-ACC (Ser 79) (**A**), p-JNK (Thr 183/Tyr 185) (**B**), p-p38 (Thr 180/Tyr 182) (**B**), p-Erk1/2(Thr 202/Tyr 204) (**B**), p-PERK (Thr 980) (**C**), p-eIF2α (Ser 51) (**C**), p-Akt (Ser 473) (**D**) and p-S6 (Ser 235/236) (**D**) were detected by Western blot using specific antibodies. Non-phosphorylated kinases and β-actin were also examined as loading controls (**A**–**D**). Blot intensity of phosphorylated kinase was quantified after normalization to non-phosphorylated kinase and was expressed as fold changes *vs.* control group (0′). Experiments were repeated three times. *p < 0.05 *vs.* control group (0′).

We first tested the effects of H_2_O_2_ on signaling changes in cultured RPE cells; Western blot results in Figure 1 demonstrated that H_2_O_2_ induced a robust and significant endoplasmic reticulum stress (ER stress), AMP activated protein kinase (AMPK) and mitogen-activated protein kinase (MAPK) signaling cascade activation. We used phosphorylation of PERK (RNA-dependent-protein-kinase-like endoplasmic-reticulum kinase) and eIF2α (α-subunit of eukaryotic translation initiation factor 2) as indicators of ER stress activation (Figure 1C). AMPK activation was reflected by phosphorylation of AMPKα (Thr 172) and ACC (Acetyl-CoA Carboxylase, Ser 79) (Figure 1A), while MAPK activation was demonstrated by phosphorylation of JNK, ERK and p38 (Figure 1B). H_2_O_2_ only had a minor effect of Akt phosphorylation (Figure 1D). Notably, H_2_O_2_ reduced S6 phosphorylation, an indicator of mTORC1 (mammalian target of rapamycin complex 1) activation (Figure 1D). These results together suggest that H_2_O_2_ activates ER stress, AMPK and MAPK signal cascades, while inhibiting mTOR1 activation in cultured RPE cells (Figure 1).

### 2.2. H_2_O_2_ Induces an Early Ceramide Production, Inhibited by Fumonisin B1

**Figure 2:**
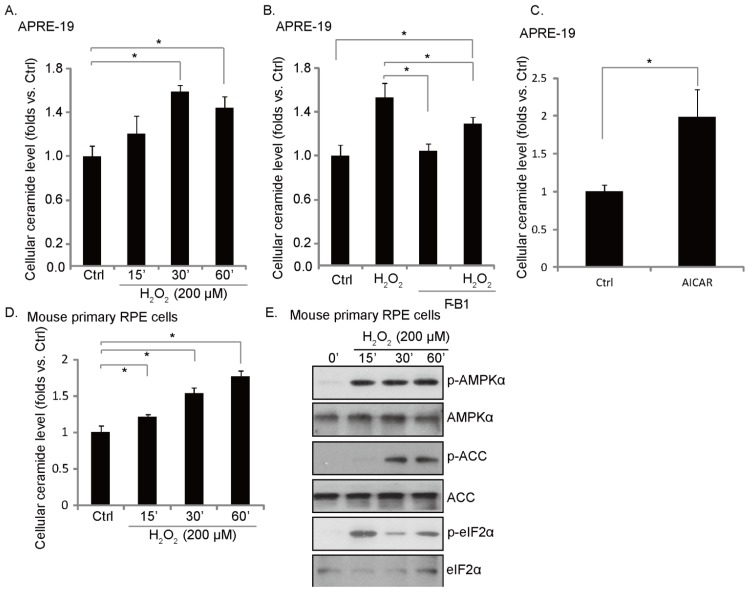
H_2_O_2_ induces an early ceramide production, inhibited by fumonisin B1. APRE-19 cells (**A**,**C**) or primary mouse RPE cells (**D**,**E**) were either left untreated or treated with H_2_O_2_ (200 μM) or 5-amino-1-β-D-ribofuranosyl-imidazole-4-carboxamide (AICAR) (1 mM) or indicated time points; cellular ceramide production was analyzed, quantified and was expressed as fold changes *vs.* untreated control group; phospho- and non-phospho- AMPK, ACC and eIF2α were detected as described above (**E**); (**B**) APRE-19 cells were pretreated with ceramide *de novo* synthase inhibitor fumonisin B1 (10 μM) for one hour, followed by H_2_O_2_ (200 μM) for indicated time points; cellular ceramides production was analyzed, quantified and expressed as fold changes *vs.* control group (Ctrl). Experiments were repeated three times. *p < 0.05.

It has been shown that H_2_O_2_ stimulation induces ceramide-dependent cell apoptosis [17]. Results in Figure 2A demonstrated that H_2_O_2_ stimulation induced a fast ceramide production in cultured RPE cells. Fumonisin B1 (F-B1), a ceramide *de novo* synthase inhibitor [18,19], suppressed ceramide induction by H_2_O_2_ (Figure 2B). These results suggest that H_2_O_2_ induces ceramide production through a *de novo* synthesis pathway, which might be important for RPE cell apoptosis (see below). Interestingly, AICAR, the AMPK activator, also induced ceramide production (Figure 2C), indicating that ceramide production might be associated with AMPK activation in H_2_O_2_-treated cells (Figure 2C). Meanwhile, a transit ceramide production (Figure 2D) and a robust AMPK activation (Figure 2E) were also seen in H_2_O_2_-treated primary mouse RPE cells.

### 2.3. H_2_O_2_-Induced ER Stress and AMPK Activation Is Inhibited by Salubrinal (Sal), but Enhanced by C6 Ceramide

**Figure 3:**
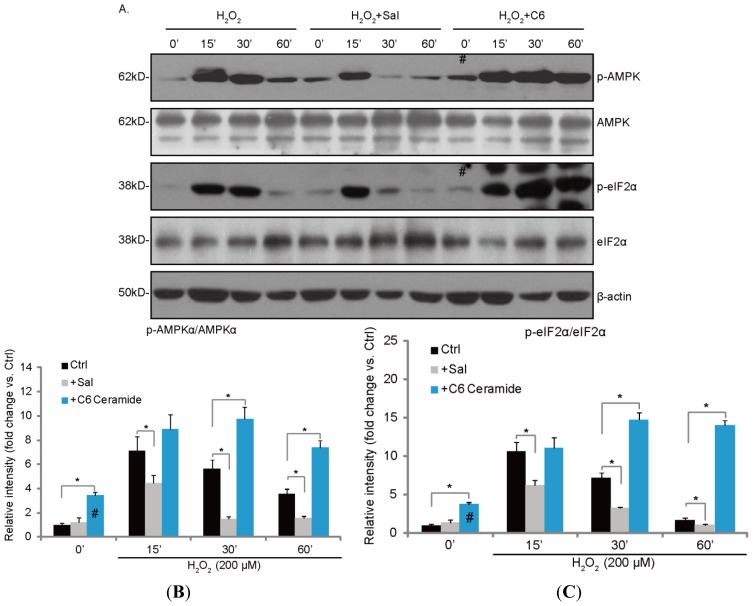
H_2_O_2_-induced AMPK and ER stress is inhibited by salubrinal (Sal), but enhanced by C6 ceramide. APRE-19 cells were pretreated with Sal (10 μM) or C6 ceramide (10 μg/mL) for 1 h, followed by H_2_O_2_ (200 μM) stimulation for indicated time points; the phosphorylation of AMPKα (Thr 172) and eIF2α (Ser 51) were detected by Western-blots. β-actin, non-phospho- AMPK and eIF2α were also examined (**A**); Blot intensity of p-AMPK and p-eIF2α was quantified after normalization to non-phospho-kinases and expressed as fold changes *vs.* control group (Ctrl) (**B**,**C**). Experiments were repeated three times. *p < 0.05. (**B**) (**C**)

We then tested the possible involvement of ceramide in H_2_O_2_-induced AMPK and ER stress activation. As shown in Figure 3A, ER stress inhibitor salubrinal (Sal) significantly reduced H_2_O_2_-induced AMPK activation (Figure 3A,B) and eIF2α phosphorylation (Figure 3A,C). Notably, short-chain cell permeable C6 ceramide alone also promoted AMPK (# in Figure 3A,B) and eIF2α phosphorylation (# in Figure 3A,C). Further, C6 ceramide significantly enhanced AMPK and ER stress activation by H_2_O_2_ in cultured RPE cells (Figure 3A–C). These results suggest that H_2_O_2_-induced ceramide production might be required for ER stress and AMPK activation (Figure 3).

### 2.4. H_2_O_2_-Induced RPE Cell Apoptosis Is Suppressed by Ceramide-ER Stress-AMPK Inhibitors

**Figure 4:**
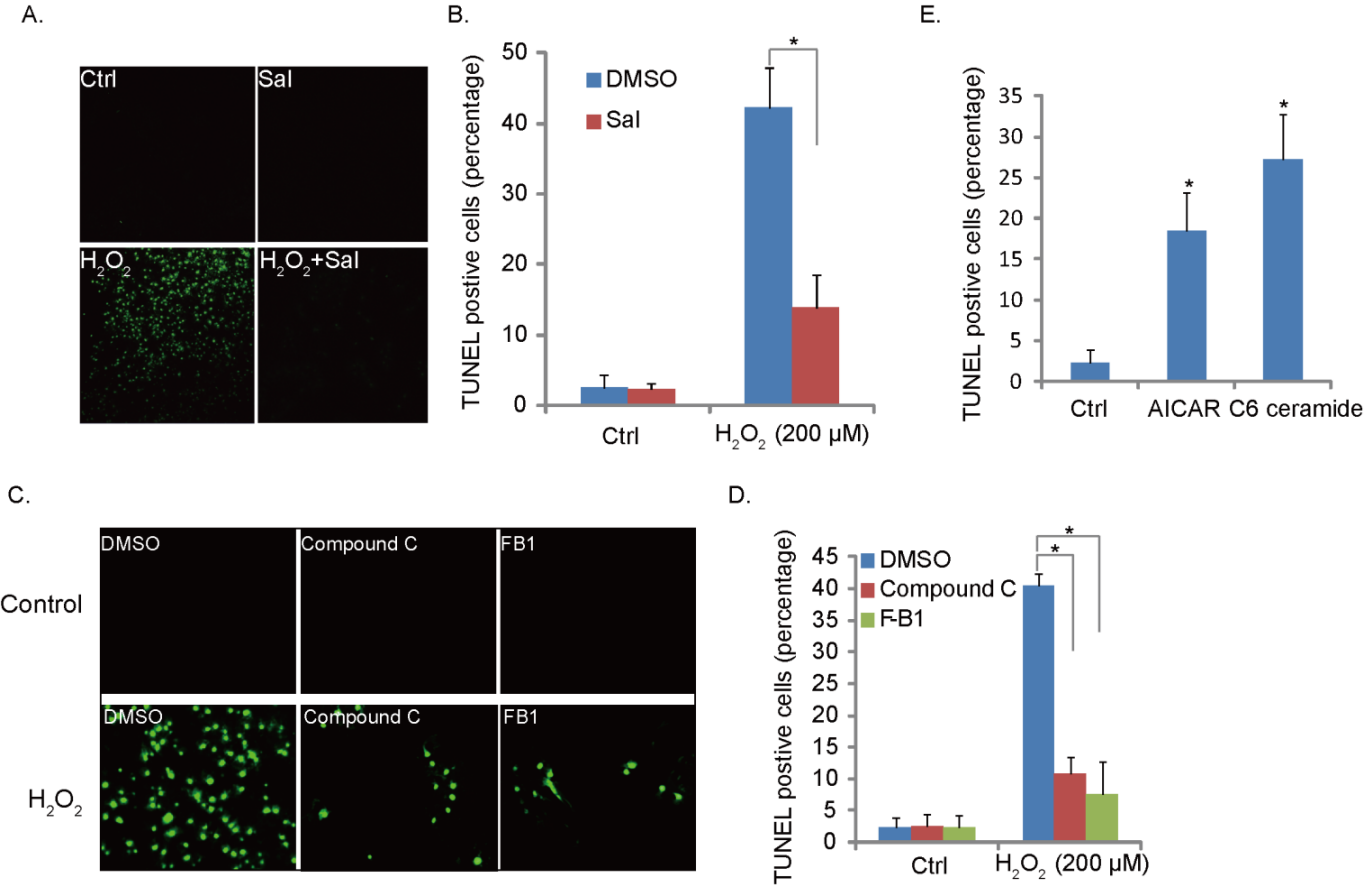
H_2_O_2_-induced RPE cell apoptosis is suppressed by ceramide-ER stress-AMPK inhibitors. APRE-19 cells were pretreated with Sal (10 μM) for one hour, followed by H_2_O_2_ (200 μM) for 24 h; cell apoptosis was detected by TUNEL staining (**A**,**B**); Cultured APRE-19 cells (RPE cells) were treated as follows: control (Ctrl), H_2_O_2_ (200 μM), fumonisin B1 (F-B1, 10 μM), compound C (10 μM), fumonisin B1 + H_2_O_2_, compound C + H_2_O_2_ (**C**,**D**), AICAR (1 mM) and C6-ceramide (10 μg/mL) (**E**); cell apoptosis was detected by TUNEL staining. Experiments were repeated three times. *p < 0.05.

We have characterized H_2_O_2_-induced ceramide-ER stress-AMPK signaling; we then tested the potential role of this signaling pathway in H_2_O_2_-induced RPE cell apoptosis. TUNEL staining was applied to test the apoptosis of RPE cells. Results in Figure 4A-B demonstrated that ER stress inhibitor Sal significantly inhibited H_2_O_2_-induced RPE cell apoptosis, indicating that ER stress is pro-apoptotic in our model. Further, AMPK inhibitor compound C and ceramide synthase inhibitor fumonisin B1 (F-B1) both suppressed H_2_O_2_-induced RPE cell apoptosis (Figure 4C,D); these results suggest that ceramide-ER stress-AMPK signaling pathway is important for H_2_O_2_-induced cell apoptosis. To further support this, we found that AMPK activator AICAR and C6 ceramide both promoted RPE cell apoptosis (Figure 4E). Together, we propose that H_2_O_2_ activates ceramide-ER stress-AMPK signaling to promote RPE cell apoptosis.

### 2.5. UV Induces Ceramide Production, ER Stress/AMPK Activation and RPE Cell Death

**Figure 5:**
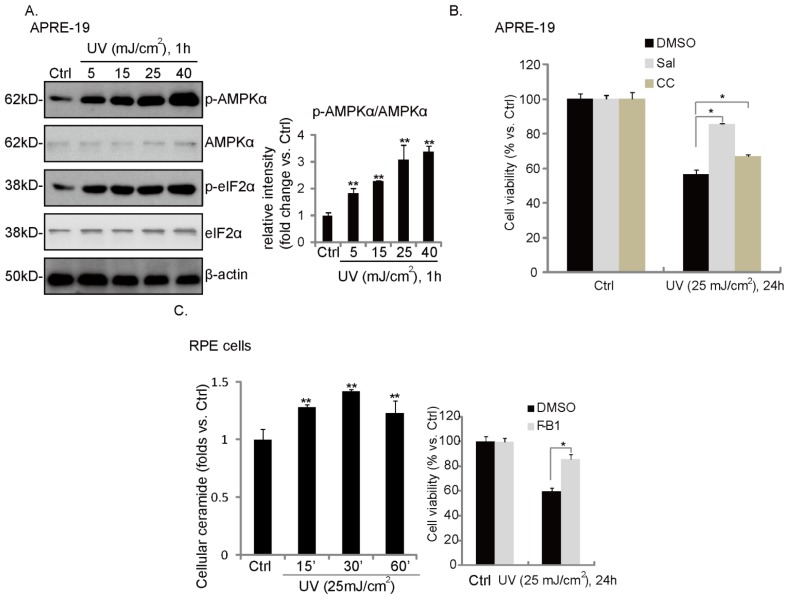
UV induces ceramide production, ER stress/AMPK activation and RPE cell death. APRE-19 cells were irradiated with indicated dosage of UV; afterwards, cells were further incubated in culture medium for one hour. Phospho- and non-phospho-eIF2α/AMPKα, as well as β-actin were tested (**A**); APRE-19 cells were irradiated with UV (25 mJ/cm^2^); afterwards, cell were incubated in culture medium for an additional 15, 30 and 60 min; cellular ceramide level was examined (**B**, **left panel**); APRE-19 cells were pretreated with fumonisin B1 (F-B1, 10 μM) (**B**, **right panel**), Sal (10 μM) or compound C (10 μM) for 1 h, followed by UV (25 mJ/cm^2^) radiation. Cells were then cultured in culture medium for an additional 24 h; cell viability was examined by MTT assay (**C**). Experiments were repeated three times. ** *p* < 0.05 *vs.* untreated control group (C, left panel), *p < 0.05 (B,C, right panel).

**Figure 6:**
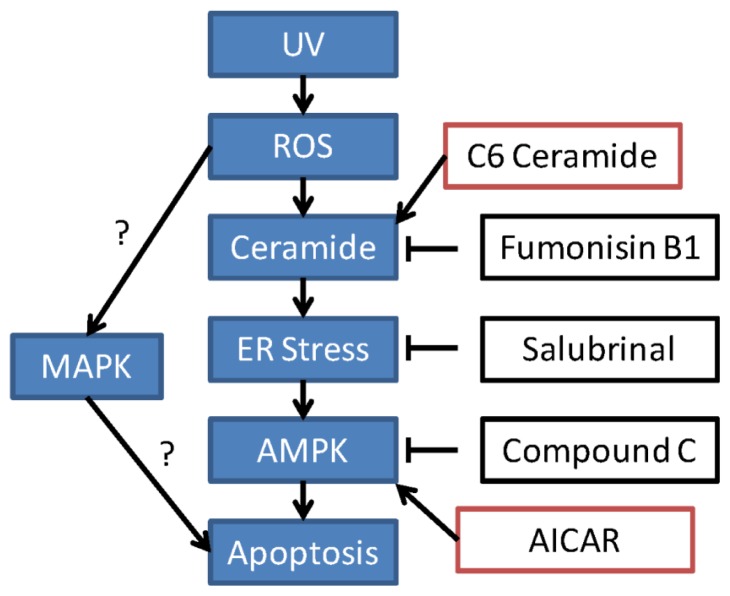
The proposed signaling pathway of this study. In RPE cells, UV radiation induces ROS production to induce an early ceramide production. Increased ceramide activates ER stress, which serves as an upstream signaling for AMPK activation. AMPK activation appears to be pro-apoptotic in this system. Suppression of this signaling axis by ceramide synthase inhibitor fumonisin B1, ER stress inhibitor salubrinal or by AMPK inhibitor compound C inhibits UV or H_2_O_2_-induced RPE cell death, while C6 ceramide and AMPK activator AICAR mimicked UV/H_2_O_2_’s effect. The role of MAPK activation in UV or H_2_O_2_-induced RPE cell death needs further investigation; also, the mechanism link between these pathways warrants more studies.

We then examined the similar signaling events in UV-treated RPE cells. Western blot results in Figure 5A showed that UV induced a significant eIF2α and AMPK phosphorylation in cultured RPE cells. Further, the cellular ceramide level was also increased after UV radiation (Figure 5B). Ceramide synthase inhibitor fumonisin B1 (F-B1) (Figure 5B, right panel), ER stress inhibitor Sal and AMPK inhibitor compound C (CC) (Figure 5C) reduced UV-induced RPE cell death, suggesting that these signal events may also be involved in UV-induced RPE cell death; see proposal signaling pathway cartoon in Figure 6.

## 3. Discussion

Under normal conditions, the endoplasmic reticulum (ER) regulates the synthesis, initial post-translational modification, proper folding and maturation of newly synthesized proteins. Meanwhile, ER is also important to maintain intracellular calcium homeostasis. The normal functions of ER are disrupted when cells face various stress conditions (ER stress) [20,21]; meanwhile, stressed cells respond to ER stress by following certain mechanisms: (a) to enhance the expression of ER chaperones and folding enzymes, such as C/EBP homologous protein (CHOP); (b) to suppress further misfolded proteins accumulation; and (c) to eliminate misfolded proteins accumulated inside the ER [22]. Although short-term and mild ER stress is generally known as a pro-survival reaction, prolonged or severe unsolved ER stress promotes cell apoptosis [22]. Previous studies have shown that UV radiation induces eIF2α phosphorylation and ER stress activation to regulate protein translation and cell apoptosis [23]. The upstream signal causing eIF2α phosphorylation by UV is not fully addressed; also, GCN2 (general control non-depressible-2) and PERK (RNA-dependent-protein-kinase-like endoplasmic-reticulum kinase) have been proposed [24,25]. In the cultured RPE cells, we here found a significant eIF2α phosphorylation after UV radiation and H_2_O_2_ treatment. The fact that ER stress inhibitor Sal inhibited H_2_O_2_-induced RPE cell apoptosis suggests that ER stress contributes to H_2_O_2_-induced RPE cell apoptosis.

We observed an early and robust ceramide production in H_2_O_2_- and UV-treated RPE cells. Significantly, H_2_O_2_-induced eIF2α phosphorylation and, following RPE cell apoptosis, was inhibited by ceramide synthase inhibitor fumonisin B1, but enhanced by the short-chain cell permeable C6 ceramide. Further, C6 ceramide by itself also induced eIF2α phosphorylation and RPE cell apoptosis. These data together indicate that UV- or H_2_O_2_-induced ER stress activation may involve the early ceramide synthesis. More direct evidence to further support this proposal is needed.

Different groups have indicated that AMP-activated protein kinase (AMPK) is an important regulator for cell apoptosis [26]. Activation of AMPK promotes cell apoptosis (see review in [27]) by regulating its downstream signal targets, including JNK [28], p53 [29] and mTOR [30]. UV [16] and ROS (H_2_O_2_) [30] are known to activate the AMPK-dependent cell apoptosis pathway. Consistent with these studies, our results here suggest that AMPK might also be important for UV- and H_2_O_2_-induced RPE cell death. However, how H_2_O_2_ activates AMPK or the potential upstream signaling for UV/H_2_O_2_-induced AMPK activation is still not fully addressed. However, groups have proposed ATM (ataxia telangiectasia mutated), CaMKKII (calmodulin-dependent protein kinase kinase II) and mitochondrial dysfunction as potential upstream kinases for AMPK activation [31]. Here, we propose that early ceramide production and ER stress might be involved in AMPK activation by H_2_O_2_, as ER stress inhibitor Sal significantly inhibited AMPK activation by H_2_O_2_, while C6 ceramide promoted AMPK activation. Our observations here are consistent with the recent study by Ji *et al.*, who identified that cell permeable ceramide C6 induces the AMPK dependent cell apoptosis pathway in multiple cancer cells [14].

AMPK inhibits mTOR complex 1 (mTORC1) activation through the two mechanisms: by phosphorylation and activation of TSC2 (Tuberous sclerosis protein 2) [32], which in turn deactivates the Rheb GTPase [32] and inhibits mTORC1 activation or by phosphorylation and inhibition of Raptor (regulatory associated protein of mTOR) [33], a key component of mTORC1 [33]. Consistent with previous studies [30], we observed a significant inhibition of S6 phosphorylation, an indicator of mTORC1 activation, following AMPK activation in H_2_O_2_-treated RPE cells, suggesting that AMPK activation may directly inhibit mTORC1 activation after H_2_O_2_ stimulation.

## 4. Materials and Methods

### 4.1. Cell Culture

Human retinal pigment epithelial cells (ARPE-19 line) were maintained in Dulbecco’s Modified Eagle’s Medium(DMEM)/Nutrient Mixture F-12 (DMEM/F12, Gibco Life Technologies, Carlsbad, CA, USA), supplemented with 10% fetal bovine serum (FBS) (Hyclone, Shanghai, China), penicillin/streptomycin (1:100, Sigma, St. Louis, MO, USA) and 4 mM L-glutamine and 0.19% HEPES (Sigma), in a humidified incubator at 37 °C and 5% CO_2_. Primary mouse RPE cell isolation and culture: C57/B6 mice at age of 3–5 days were anesthetized by 75% alcohol, and the eyeballs in asepsis were taken out and diluted several times with D-hank’s fluid. After soaking in the DMEM/F-12 (Hyclone Co., Logan, UT, USA) for 6–10 h, the eyeballs were taken out, and the retinas were striped carefully. Zero-point-one-two-five-percent parenzyme was added to digest for 20 min at 37 °C before adding culture medium (Minghai, Lanzhou, China) containing blood serum to terminate digestion. Then, the supernatant was centrifuged twice at 1000 r/min in the culture medium (80% DMEM/F-12, 20% fetal serum) to produce a cell suspension after inoculation into the 75 cm^2^ culture flask. Cells were divided to 1:2 until the cells grew identical in shape. The cells at passage 3 were used for future experiments.

### 4.2. Reagents and Chemicals

Compound C, 5-amino-1-β-D-ribofuranosyl-imidazole-4-carboxamide (AICAR) and salubrinal (Sal), were purchased from Calbiochem (Darmstadt, Germany), C6 ceramide was a gift from Dr. Zhigang Bi at Nanjing Medical University; all phosphorylation and non-phosphorylation kinases antibodies used in this study were obtained from Cell Signaling Tech (Danvers, MA, USA). Fumonisin B1 and mouse mono-clone antibody against β-actin was purchased from Sigma (St. Louis, MO, USA).

### 4.3. TUNEL Staining and Counting

RPE cell apoptosis was detected by the TUNEL (terminal deoxynucleotidyl transferase dUTP nick-end labeling) *In Situ* Cell Death Detection Kit (Roche Molecular Biochemicals, Indianapolis, IN, USA), according to the manufacturer’s instructions. RPE cells were also stained with 4′,6′-diamino-2-phenylin-dole (DAPI, blue fluorescence; Molecular Probes) to visualize the cell nuclear. The apoptosis rate was determined by TUNEL percentage, which was calculated by the number of TUNEL-positive cells, divided by the number of TUNEL-stained cells. At least 1,000 total cells in 10 views from 10 repeat wells (1 × 100) of each condition were included for counting TUNEL-positive cells, and the average was calculated.

### 4.4. Cell Viability Assay

RPE cell viability was measured by the 3-[4,5-dimethylthylthiazol-2-yl]-2,5 diphenyltetrazolium bromide (MTT) method. Briefly, the cells were collected and seeded in 96-well plate at a density of 2 × 10 ^5^ cells/cm^2^. Different seeding densities were optimized at the beginning of the experiments. After overnight incubation, cells were exposed to UV radiation or fresh medium containing indicated reagents at 37 °C. After incubation for a different period time, 20 μL of MTT tetrazolium (Sigma, St. Louis, MO, USA) salt dissolved in Hank’s balanced salt solution at a concentration of 5 mg/mL was added to each well and incubated in a CO_2_ incubator for an additional 4 h. Finally, the medium was aspirated from each well, and 150 μL of DMSO (Sigma, St. Louis, MO, USA) was added to dissolve formazan crystals. The absorbance of each well was obtained using a Dynatech MR5000 plate reader at a test wavelength of 490 nm with a reference wavelength of 630 nm.

### 4.5. Measurement of Cellular Ceramide Levels

Similar to previously reported in [34], the total pool of sphingolipids in RPE cells with indicated treatment/s were radio-labeled with 3 μCi/mL [^3^H]l-serine (30 Ci/mmol; Amersham), a precursor for sphingolipid biosynthesis. The medium was removed, and the cells were fixed in ice-cold CH_3_OH, followed by lipid extraction from the cells [35]. Aliquots of the lipid extracts were taken for the determination of the total amount of lipid-incorporated radioactivity. Acyl glycerolipids were hydrolyzed during one hour of incubation at 37 °C in CHCl_3_/CH_3_OH (1:1, *v*/*v*) containing 0.1 M KOH. The remaining lipids were re-extracted and applied on high performance thin-layer chromatography plates. Plates were developed in CHCl_3_/CH_3_OH/H_2_O (14:6:1, *v*/*v*) in the first dimension and in CHCl_3_/CH_3_COOH (9:1, *v*/*v*) in the second dimension to resolve ceramide. Ceramide-containing spots were scraped and subjected to scintillation counting. The RPE cellular ceramide level was expressed as a fold change of the untreated control group.

### 4.6. Western Blots Analysis

After indicated treatment, aliquots of 30 μg of lysed protein from each sample (lysed by 40 mM HEPES [pH 7.5], 120 mM NaCl, 1 mM EDTA, 10 mM pyrophosphate, 10 mM glycerophosphate, 50 mM NaF, 0.5 mM orthovanadate, EDTA-free protease inhibitors [Roche] and 1% Triton) from each sample was separated by 10% SDS polyacrylamide gel electrophoresis and transferred onto a polyvinylidene difluoride (PVDF) membrane (Millipore, Bedford, MA, USA). After blocking with 10% instant non-fat dry milk for one hour, membranes were incubated with specific antibodies overnight at 4 °C, followed by incubation with secondary antibodies for 45 min to one hour at room temperature. The Western blot results were visualized by an ECL machine. The intensity of each blot was quantified using ImageJ software after normalization to corresponding loading controls.

### 4.7. Statistical Analysis

Individual culture dishes or wells were analyzed separately (no pooling of samples was used). In each experiment, a minimum of three wells/dishes of each treatment were used. Each experiment was repeated a minimum of three times. In each experiment, the mean value of the repetitions was calculated, and this value was used in the statistical analysis. All data were normalized to control values of each assay and are presented as the mean ± standard deviation (SD). Data were analyzed by one-way ANOVA, followed by a Scheffe’s f-test by using SPSS software (SPSS Inc., Chicago, IL, USA). Significance was chosen as *p* < 0.05.

## 5. Conclusion

In summary, these above results suggest that UV and H_2_O_2_ activate the ceramide-ER stress-AMPK signaling axis to promote RPE cell apoptosis (see proposed signaling cartoon in Figure 6).

## Abbreviations

AMD: age-related macular degeneration
Sal: salubrinal
H_2_O_2_: hydrogen peroxide
AICAR: 5-amino-1-β-D-ribofuranosyl-imidazole-4-carboxamide
UV: ultraviolet
MTT: 3-[4,5-dimethylthylthiazol-2-yl]-2,5 diphenyltetrazolium bromide
NGF: nerve growth factor
RPE: retinal pigment epithelium
ROS: reactive oxygen species
mTOR: mammalian target of rapamycin
AMPK: AMP activated protein kinase
MAPK: mitogen-activated protein kinase
ER stress: endoplasmic reticulum stress
eIF2α: α-subunit of eukaryotic translation initiation factor 2
TUNEL: terminal deoxynucleotidyl transferase dUTP nick-end labeling.
